# Study of the Differential Activity of Thrombin Inhibitors Using Docking, QSAR, Molecular Dynamics, and MM-GBSA

**DOI:** 10.1371/journal.pone.0142774

**Published:** 2015-11-24

**Authors:** Karel Mena-Ulecia, William Tiznado, Julio Caballero

**Affiliations:** 1 Departamento de Química, Facultad de Ciencias Exactas, Universidad Andres Bello, Avenida República 252, Santiago, Chile; 2 Centro de Bioinformática y Simulación Molecular, Facultad de Ingeniería, Universidad de Talca, 2 Norte 685, Casilla 721, Talca, Chile; Wake Forest University, UNITED STATES

## Abstract

Non-peptidic thrombin inhibitors (TIs; 177 compounds) with diverse groups at motifs P_1_ (such as oxyguanidine, amidinohydrazone, amidine, amidinopiperidine), P_2_ (such as cyanofluorophenylacetamide, 2-(2-chloro-6-fluorophenyl)acetamide), and P_3_ (such as phenylethyl, arylsulfonate groups) were studied using molecular modeling to analyze their interactions with S_1_, S_2_, and S_3_ subsites of the thrombin binding site. Firstly, a protocol combining docking and three dimensional quantitative structure–activity relationship was performed. We described the orientations and preferred active conformations of the studied inhibitors, and derived a predictive CoMSIA model including steric, donor hydrogen bond, and acceptor hydrogen bond fields. Secondly, the dynamic behaviors of some selected TIs (compounds **26**, **133**, **147**, **149**, **162**, and **177** in this manuscript) that contain different molecular features and different activities were analyzed by creating the solvated models and using molecular dynamics (MD) simulations. We used the conformational structures derived from MD to accomplish binding free energetic calculations using MM-GBSA. With this analysis, we theorized about the effect of van der Waals contacts, electrostatic interactions and solvation in the potency of TIs. In general, the contents reported in this article help to understand the physical and chemical characteristics of thrombin-inhibitor complexes.

## Introduction

Thromboembolic diseases are among the principal causes of mortality in the world. Vein thrombosis can progress to pulmonary embolism. These disorders, identified with the term venous thromboembolism (VTE), affect several million people around the world [1]. VTE is the third leading cause of cardiovascular-related death, after myocardial infarction and stroke [2].

The central role of the serine protease thrombin in thrombosis and haemostasis makes it an attractive target for antithrombotic therapy [3]. Thrombin catalyzes the conversion of soluble fibrinogen to insoluble fibrin in the clotting cascade, and also acts on other substrates such as factor V, factor VIII, factor XI, and factor XIII. It is widely believed that an oral thrombin inhibitor (TI) could provide a new standard of care in anticoagulation therapy.

The discovery of small molecule TIs is an important goal for anti-thrombotic therapy [4]. In the last years, potent and selective inhibitors have been reported, such as pyridones, acetamides, oxyguanidines, aminopiperidines, amidines, and amidinohydrazones [5–13]. These sets have shown that subtle structural differences in compounds (due to the presence of similar scaffolds or the same scaffolds with different substituents) can lead to big differences in their thrombin inhibitory activities.

The knowledge of the relevant structural features that positively influence the activity of TIs is important for the design of potent compounds. Molecular modeling has demonstrated to be a powerful support to investigate bioactive compounds and their structure-activity relationship (SAR) with the main purpose of identifying the molecular features that contribute to a high bioactivity. These methods have been applied for studying TIs. Several quantitative structure-activity relationship (QSAR) models were reported using approaches such as classic QSAR [14], CoMFA/CoMSIA [15,16], topological descriptors [17], and artificial neural networks [18]. Other reports used docking and molecular dynamics (MD) simulations to study structural features of several TIs identified wit a high activity [19–21]. In general, these reports do not include an analysis of the interactions between inhibitors and different subsites of the thrombin binding site. An exception is the work of Nilsson et al. [22]. These authors designed a set of compounds to bind to the S_2_ and S_3_ subsites with no interactions in S_1_, and developed a classic QSAR model to study the chemical features that are important for the prediction of their binding constants. Other exception is the work of Bhunia et al. [23], which used three dimensional (3D) QSAR and MD simulations to profile structural determinants for the selectivity of representative diverse classes of thrombin-selective inhibitors.

In the current work, we applied some of the popular molecular modeling methods to study the interactions of TIs with S_1_, S_2_, and S_3_ subsites of the thrombin binding site. We studied the orientations and SAR for 177 TIs by using a protocol that includes docking and the 3D-QSAR method CoMSIA. Additionally, we analyzed the dynamical behavior for some selected compounds (**26**, **133**, **147**, **149**, **162**, and **177**) by using MD and free energy calculations. By means of a comparison of the selected systems, we gained further insight into the role played by different TI molecular constituents in the binding affinities due to interactions with different subsites in the thrombin active site.

## Materials and Methods

### Data set

**Fig 1:**
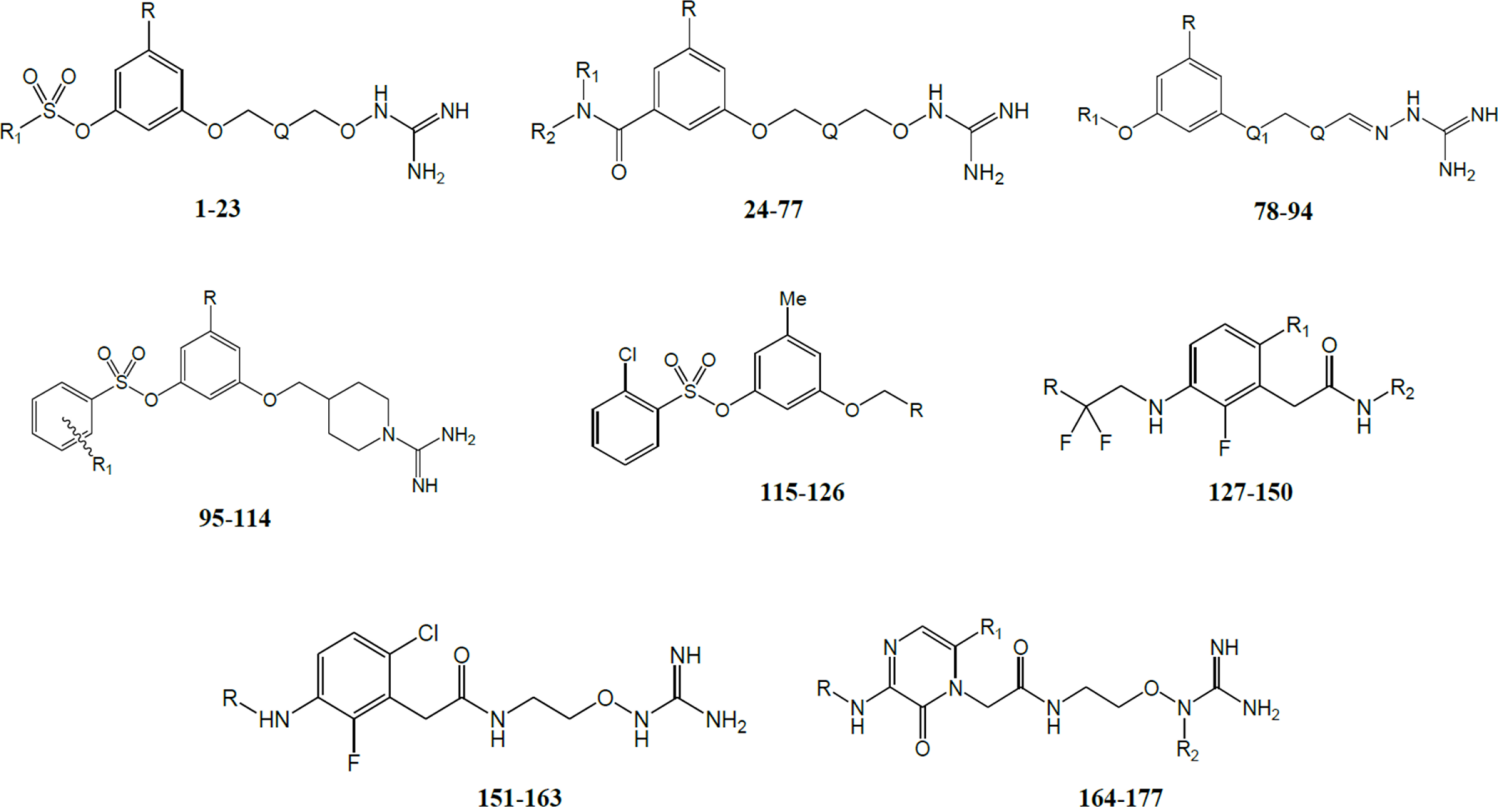
Structures of TIs.

The primary structures and activities of 177 TIs were taken from the literature [5–13]. Inhibitory activities were collected and transformed into log(10^3^/Ki) values. Ki values are in nM and represent the enzyme inhibition constants. TI structures are in Fig 1 and their biological activities used in this study are summarized in Table 1. The chemical structures were sketched using the molecular editor of Maestro 9.0 software suite [24].

**Table 1:** Experimental and predicted thrombin inhibitory activities (log(10^3^/Ki) (nM)) using CoMSIA-SDA model.

Compounds **1**–**23**			
compound	R, R_1_, R_2_, Q	Exp. Log(10^3^/Ki)	Calc. Log(10^3^/Ki)
**1**	R = Me; R_1_ = 2-Cl phenyl; Q = CH_2_	2.68	2.12
**2**	R = Me; R_1_ = 2-CN phenyl; Q = CH_2_	2.00	1.56
**3**	R = Me; R_1_ = 2-OMe phenyl; Q = CH_2_	2.17	2.10
**4**	R = Me; R_1_ = 2-NH_2_ phenyl; Q = CH_2_	1.06	1.40
**5** ^**a**^	R = Me; R_1_ = 2-(MeSO_2_)Ph; Q = CH_2_	1.92	-
**6**	R = Me; R_1_ = 2-(PheSO_2_)Ph; Q = CH_2_	2.32	2.84
**7**	R = Me; R_1_ = 3-(MeSO_2_)Ph; Q = CH_2_	0.69	0.73
**8**	R = Me; R_1_ = 2,4-(MeSO_2_)_2_ Ph; Q = CH_2_	0.13	0.14
**9**	R = Me; R_1_ = 8-quinolinyl; Q = CH_2_	2.17	1.77
**10**	R = Me; R_1_ = 5-isoquinolinyl; Q = CH_2_	1.88	2.05
**11**	R = Me; R_1_ = 1,2,3,4-tetrahydro-8-quinolinyl; Q = CH_2_	1.51	1.37
**12**	R = Me; R_1_ = 1,1-dioxido-2,3-dihydro-1-benzothien-6-yl; Q = CH_2_	0.99	0.82
**13**	R = Me; R_1_ = 2-(MeSO_2_)Ph; Q = C(–C_2_H_4_–)	2.11	2.06
**14**	R = Me; R_1_ = 2-(MeSO_2_)Ph; Q = CHOH	0.86	0.71
**15**	R = Me; R_1_ = 2-(MeSO_2_)Ph; Q = CHF	0.94	0.83
**16**	R = Me; R_1_ = 2-(MeSO_2_)Ph; Q = CF_2_	0.44	0.68
**17**	R = Me; R_1_ = 2-(MeSO_2_)Ph; Q = C = CH_2_	1.49	1.20
**18** ^**a**^	R = Me; R_1_ = 2-(MeSO_2_)Ph; Q = bond	-0.42	-
**19**	R = OMe; R_1_ = 2-(MeSO_2_)Ph; Q = CH_2_	1.46	1.49
**20**	R = Et; R_1_ = 2-(MeSO_2_)Ph; Q = CH_2_	1.53	1.91
**21**	R = Cl; R_1_ = 2-(MeSO_2_)Ph; Q = CH_2_	1.41	1.40
**22**	R = CH_2_OH; R_1_ = 2-(MeSO_2_)Ph; Q = C(–C_2_H_4_–)	0.40	0.43
**23**	R = F; R_1_ = 2-(MeSO_2_)Ph; Q = C(–C_2_H_4_–)	1.31	1.34
Compounds **24**–**77**			
compound	R, R_1_, R_2_, Q	Exp. Log(10^3^/Ki)	Calc. Log(10^3^/Ki)
**24**	R = Cl; R_1_ = allyl; R_2_ = cyclopentyl; Q = CH_2_	1.68	1.55
**25**	R = Cl; R_1_ = Me; R_2_ = cyclohexyl; Q = CH_2_	1.48	1.62
**26** ^**b**^ ^**,**^ ^**c**^	R = Cl; R_1_ = Me; R_2_ = cyclopentyl; Q = CH_2_	0.43	1.46
**27**	R = Cl; R_1_ = Pr; R_2_ = cyclopentylmethyl; Q = CH_2_	1.42	1.66
**28**	R = Cl; R_1_ = Pr; R_2_ = cyclobutylmethyl; Q = CH_2_	1.80	1.87
**29** ^**b**^	R = Cl; R_1_ = Pr; R_2_ = cyclopropylmethyl; Q = CH_2_	1.64	1.57
**30** ^**b**^	R = Cl; R_1_ = 2-carboxyethyl; R_2_ = cyclopropylmethyl; Q = CH_2_	0.67	1.61
**31**	R = Cl; R_1_ = 3-methoxy-3-oxopropyl; R_2_ = cyclopropylmethyl; Q = CH_2_	1.42	1.79
**32**	R = Cl; R_1_ = 2-methoxyethyl; R_2_ = cyclopropylmethyl; Q = CH_2_	0.81	0.58
**33** ^**a**^	R = Cl; R_1_ = 2-(1-pyrrolidinyl)ethyl; R_2_ = cyclopropylmethyl; Q = CH_2_	0.69	-
**34** ^**b**^	R = Cl; R_1_ = 2-(4-morpholinyl)ethyl; R_2_ = cyclopropylmethyl; Q = CH_2_	0.80	1.45
**35**	R = Cl; R_1_ = 2-(2-pyridinyl)ethyl; R_2_ = cyclopropylmethyl; Q = CH_2_	1.23	1.13
**36**	R = Cl; R_1_ = 2-(3-pyridinyl)ethyl; R_2_ = cyclopropylmethyl; Q = CH_2_	1.33	1.37
**37**	R = Cl; R_1_ = 2-(4-pyridinyl)ethyl; R_2_ = cyclopropylmethyl; Q = CH_2_	1.28	1.33
**38**	R = Cl; R_1_ = allyl; R_2_ = 3-furylmethyl; Q = CH_2_	1.70	1.16
**39**	R = Cl; R_1_ = 3-furylmethyl; R_2_ = cyclopropylmethyl; Q = CH_2_	1.77	1.73
**40**	R = Cl; R_1_ = allyl; R_2_ = 2-thienylmethyl; Q = CH_2_	0.83	0.94
**41**	R = Cl; R_1_ = Et; R_2_ = 4-pyridinylmethyl; Q = CH_2_	-0.20	-0.34
**42**	R = Cl; R_1_ = Et; R_2_ = Bn; Q = CH_2_	1.04	1.68
**43**	R = Cl; R_1_, R_2_ = –(CH_2_)_5_–; Q = CH_2_	0.06	0.60
**44**	R = Cl; R_1_, R_2_ = –CH_2_-CH_2_-O-CH_2_-CH_2_–; Q = CH_2_	-0.88	-1.25
**45**	R = Cl; R_1_, R_2_ = –(CH_2_)_6_–; Q = CH_2_	0.33	0.73
**46**	R = Cl; R_1_, R_2_ = –CH_2_-CH_2_-CH(Bn)-CH_2_-CH_2_–; Q = CH_2_	-0.18	0.40
**47**	R = Cl; R_1_, R_2_ = –CH_2_-CH_2_-N(Ph)-CH_2_-CH_2_–; Q = CH_2_	-0.46	-0.36
**48**	R = Cl; R_1_, R_2_ = –CH_2_-CH_2_-N(2-pyridinyl)-CH_2_-CH_2_–; Q = CH_2_	0.19	0.23
**49**	R = Cl; R_1_, R_2_ = –CH_2_-C(–C_4_H_4_–)C-CH_2_-CH_2_–; Q = CH_2_	0.77	1.03
**50**	R = Me; R_1_ = allyl; R_2_ = cyclopentyl; Q = CH_2_	2.00	1.56
**51** ^**b**^	R = Me; R_1_ = Pr; R_2_ = cyclobutylmethyl; Q = CH_2_	1.77	1.74
**52**	R = Me; R_1_ = Et; R_2_ = cyclohexyl; Q = CH_2_	2.22	2.04
**53**	R = Me; R_1_ = 6-amino-6-oxohexyl; R_2_ = cyclohexyl; Q = CH_2_	2.10	1.86
**54**	R = Me; R_1_ = allyl; R_2_ = cyclohexyl; Q = CH_2_	2.05	1.97
**55**	R = Me; R_1_ = 3-ethoxy-3-oxopropyl; R_2_ = cyclopropylmethyl; Q = CH_2_	1.19	1.09
**56** ^**b**^	R = Me; R_1_ = Pr; R_2_ = 3-furylmethyl; Q = CH_2_	2.05	1.54
**57** ^**b**^	R = Me; R_1_ = Bu; R_2_ = 3-furylmethyl; Q = CH_2_	2.10	1.57
**58**	R = Me; R_1_ = iPn; R_2_ = 3-furylmethyl; Q = CH_2_	2.16	1.99
**59**	R = Me; R_1_ = cyclopropylmethyl; R_2_ = 3-furylmethyl; Q = CH_2_	1.96	1.63
**60**	R = Me; R_1_ = iPr; R_2_ = 3-furylmethyl; Q = CH_2_	1.17	1.43
**61**	R = Me; R_1_ = cyclobutyl; R_2_ = 3-furylmethyl; Q = CH_2_	1.66	1.81
**62**	R = Me; R_1_ = cyclohexylmethyl; R_2_ = 3-furylmethyl; Q = CH_2_	1.68	1.80
**63**	R = Me; R_1_ = 2-methoxyethyl; R_2_ = 3-furylmethyl; Q = CH_2_	1.55	1.50
**64**	R = Me; R_1_ = 3-amino-3-oxopropyl; R_2_ = 3-furylmethyl; Q = CH_2_	0.96	1.18
**65**	R = Me; R_1_ = 2-(ethylsulfanyl)ethyl; R_2_ = 3-furylmethyl; Q = CH_2_	2.22	2.01
**66**	R = Me; R_1_ = Pr; R_2_ = 3-thienylmethyl; Q = CH_2_	1.32	1.43
**67**	R = Me; R_1_ = Pr; R_2_ = 3-pyridinylmethyl; Q = CH_2_	1.28	1.94
**68** ^**b**^	R = Me; R_1_ = Pr; R_2_ = 4-F bencyl; Q = CH_2_	1.68	1.37
**69**	R = Me; R_1_ = cyclopropylmethyl; R_2_ = 4-pyridinylmethyl; Q = CH_2_	1.62	1.35
**70**	R = Me; R_1_ = Pr; R_2_ = 1,3-thiazol-2-ylmethyl; Q = CH_2_	0.71	0.52
**71**	R = Me; R_1_ = cyclopropylmethyl; R_2_ = 3-thienylmethyl; Q = CH_2_	1.51	1.37
**72** ^**b**^	R = Me; R_1_ = Pr; R_2_ = 2-pyridinylmethyl; Q = CH_2_	1.03	2.29
**73**	R = Me; R_1_ = cyclopropylmethyl; R_2_ = 3-pyridinylmethyl; Q = CH_2_	1.47	1.50
**74**	R = Me; R_1_ = Pr; R_2_ = 4-pyridinylmethyl; Q = CH_2_	1.14	1.00
**75** ^**b**^	R = Me; R_1_ = cyclopropylmethyl; R_2_ = 2-pyridinylmethyl; Q = CH_2_	1.07	1.27
**76**	R = Me; R_1_ = Pr; R_2_ = 3-furylmethyl; Q = C(–C_2_H_4_–)	2.40	2.52
**77** ^**b**^	R = Me; R_1_ = cyclopropylmethyl; R_2_ = 3-furylmethyl; Q = C(–C_2_H_4_–)	2.40	0.96
Compounds **78**–**94**			
compound	R, R_1_, Q, Q_1_	Exp. Log(10^3^/Ki)	Calc. Log(10^3^/Ki)
**78**	R = Me; R_1_ = (2-Cl phenyl)SO_2_; Q = CH_2_; Q_1_ = O	2.08	1.96
**79**	R = Me; R_1_ = (2.3-dichlorophenyl)SO_2_; Q = CH_2_; Q_1_ = O	2.03	2.19
**80**	R = Me; R_1_ = (2-OMe phenyl)SO_2_; Q = CH_2_; Q_1_ = O	1.96	1.73
**81**	R = Me; R_1_ = (2-OCF_3_ phenyl)SO_2_; Q = CH_2_; Q_1_ = O	1.96	1.74
**82** ^**b**^	R = Me; R_1_ = (3-Me phenyl)SO_2_; Q = CH_2_; Q_1_ = O	2.22	1.83
**83**	R = Me; R_1_ = (2-(MeSO_2_) phenyl)SO_2_; Q = CH_2_; Q_1_ = O	1.70	1.68
**84**	R = Me; R_1_ = (2-CN phenyl)SO_2_; Q = CH_2_; Q_1_ = O	2.04	2.02
**85**	R = Me; R_1_ = (2-CF_3_ phenyl)SO_2_; Q = CH_2_; Q_1_ = O	2.36	2.23
**86** ^**b**^	R = Me; R_1_ = (5-Cl thiophen-2-yl)SO_2_; Q = CH_2_; Q_1_ = O	2.04	1.19
**87** ^**b**^	R = Me; R_1_ = (naphthalen-1-yl)SO_2_; Q = CH_2_; Q_1_ = O	1.36	1.07
**88**	R = Me; R_1_ = (3-pyridinyl)SO_2_; Q = CH_2_; Q_1_ = O	1.44	1.71
**89**	R = Me; R_1_ = (quinolin-8-yl)SO_2_; Q = CH_2_; Q_1_ = O	2.33	1.86
**90** ^**a**^	R = Me; R_1_ = 2-CF_3_ bencyl; Q = CH_2_; Q_1_ = O	0.38	-
**91**	R = OMe; R_1_ = (2-Cl phenyl)SO_2_; Q = CH_2_; Q_1_ = CH_2_	0.00	-0.17
**92** ^**b**^	R = Me; R_1_ = (2-OMe phenyl)SO_2_; Q = bond; Q_1_ = O	0.00	1.20
**93**	R = Me; R_1_ = (2-OMe phenyl)SO_2_; Q = (CH_2_)_2_; Q_1_ = O	-0.11	0.24
**94** ^**b**^	R = Me; R_1_ = (2-CN phenyl)SO_2_; Q = C(–C_2_H_4_–); Q_1_ = O	1.40	1.50
Compounds **95**–**114**			
compound	R. R_1_	Exp. Log(10^3^/Ki)	Calc. Log(10^3^/Ki)
**95** ^**b**^	R = Me; R_1_ = H	1.50	1.45
**96**	R = Me; R_1_ = 2-CF_3_	1.85	1.40
**97**	R = Me; R_1_ = 2-NO_2_	1.80	1.27
**98**	R = Me; R_1_ = 2-NH_2_	1.28	1.41
**99**	R = Me; R_1_ = 2- CO_2_Me	1.55	1.40
**100**	R = Me; R_1_ = 3-Cl	1.41	1.17
**101** ^**b**^	R = Me; R_1_ = 3-CF_3_	0.35	1.05
**102**	R = Me; R_1_ = 3-Me	1.44	1.34
**103**	R = Me; R_1_ = 3-NO_2_	0.94	0.95
**104**	R = Me; R_1_ = 3-NH_2_	1.50	1.40
**105**	R = Me; R_1_ = 2.3-diCl	1.66	1.14
**106**	R = Me; R_1_ = 4-NO_2_	0.49	0.91
**107**	R = Me; R_1_ = 4-NH_2_	0.40	0.45
**108** ^**b**^	R = Me; R_1_ = 2.3 -CH = CH-CH = CH-	1.46	1.11
**109** ^**b**^	R = H; R_1_ = 2-Cl	0.72	0.88
**110**	R = CO_2_Me; R_1_ = 2-Cl	-0.57	-0.53
**111**	R = OMe; R_1_ = 2-Cl	1.36	1.58
**112**	R = Et; R_1_ = 2-Cl	1.41	1.48
**113**	R = CH_2_OH; R_1_ = 2-Cl	0.31	0.40
**114**	R = Cl; R_1_ = 2-Cl	1.52	1.05
Compounds **115**–**126**			
compound	R	Exp. Log(10^3^/Ki)	Calc. Log(10^3^/Ki)
**115**	R = 3-amino-3-iminopropyl	0.34	0.73
**116**	R = 5-amino-5-iminopentyl	1.16	0.94
**117**	R = 4-[amino(imino)methyl]cyclohexyl	-0.32	0.27
**118** ^**b**^	R = 4-[amino(imino)methyl]phenyl	0.96	0.54
**119**	R = 3-[amino(imino)methyl]phenyl	1.00	0.77
**120**	R = 1-ethanimidoyl-4-piperidinyl	-0.11	0.85
**121**	R = [methyl(4-pyridinyl)amino]methyl	1.96	2.27
**122** ^**b**^	R = 1-[amino(imino)methyl]-4-piperidinyl	2.34	1.09
**123** ^**b**^	R = 1-[amino(imino)methyl]-3-piperidinyl	0.50	0.03
**124**	R = 3-{[amino(imino)methyl]amino}ethyl	1.48	1.23
**125** ^**b**^	R = 3-{[amino(imino)methyl]amino}propyl	1.89	1.45
**126**	R = 3-{[amino(imino)methyl]amino}butyl	0.59	0.41
Compounds **127**–**150**			
compound	R. R_1_. R_2_	Exp. Log(10^3^/Ki)	Calc. Log(10^3^/Ki)
**127**	R = phenyl; R_1_ = CN; R_2_ = 2-guanidinooxy-ethyl	2.64	3.10
**128**	R = pyridin-2-yl; R_1_ = CN; R_2_ = 2-guanidinooxy-ethyl	2.92	3.34
**129**	R = 3-methylpyridin-2-yl; R_1_ = CN; R_2_ = 2-guanidinooxy-ethyl	2.00	2.45
**130**	R = 4-methylpyridin-2-yl; R_1_ = CN; R_2_ = 2-guanidinooxy-ethyl	2.54	2.67
**131**	R = 5-methylpyridin-2-yl; R_1_ = CN; R_2_ = 2-guanidinooxy-ethyl	2.92	2.71
**132**	R = 6-methylpyridin-2-yl; R_1_ = CN; R_2_ = 2-guanidinooxy-ethyl	3.10	2.43
**133** ^**c**^	R = 5-Cl-pyridin-2-yl; R_1_ = CN; R_2_ = 2-guanidinooxy-ethyl	3.24	3.35
**134**	R = 8-quinolyl; R_1_ = CN; R_2_ = 2-guanidinooxy-ethyl	3.42	2.96
**135**	R = 3-Cl-phenyl; R_1_ = CN; R_2_ = 2-guanidinooxy-ethyl	2.77	2.73
**136**	R = 3-F-phenyl; R_1_ = CN; R_2_ = 2-guanidinooxy-ethyl	2.50	2.46
**137** ^**b**^	R = 3.4-diF-phenyl; R_1_ = CN; R_2_ = 2-guanidinooxy-ethyl	2.75	3.43
**138**	R = 2-SO_2_Me-phenyl; R_1_ = CN; R_2_ = 2-guanidinooxy-ethyl	3.19	2.94
**139** ^**b**^	R = 1-oxy-2-pyridyl; R_1_ = CN; R_2_ = 2-guanidinooxy-ethyl	2.89	3.29
**140** ^**a**^	R = 5-Cl-1-oxy-2-pyridyl; R_1_ = CN; R_2_ = 2-guanidinooxy-ethyl	3.18	-
**141**	R = pyridin-2-yl; R_1_ = CN; R_2_ = 2-methylguanidinooxy-ethyl	2.05	1.71
**142**	R = pyridin-2-yl; R_1_ = CN; R_2_ = (6-aminopyridin-3-yl)methyl	1.96	2.30
**143**	R = pyridin-2-yl; R_1_ = CN; R_2_ = (6-amino-2-methylpyridin-3-yl)methyl	3.11	2.87
**144**	R = pyridin-2-yl; R_1_ = CN; R_2_ = (3-fluoropyridin-2-yl)methyl	1.48	2.06
**145**	R = pyridin-2-yl; R_1_ = CN; R_2_ = (3-aminobenzo[d]isoxazol-6-yl)methyl	1.72	1.57
**146**	R = Ph; R_1_ = Cl; R_2_ = (6-NH_2_-2-Me-3-pyridyl)methyl	1.99	2.54
**147** ^**c**^	R = Ph; R_1_ = Cl; R_2_ = (6- NH_2_-2.4-dimethyl-3-pyridyl)methyl	2.48	2.45
**148**	R = 4-F-1-naphthyl; R_1_ = Cl; R_2_ = (6-NH_2_-2-Me-3-pyridyl)methyl	2.55	2.54
**149** ^**c**^	R = 5-Cl-2-pyridyl; R_1_ = Cl; R_2_ = (6-NH_2_-2-Me-3-pyridyl)methyl	3.16	2.69
**150**	R = 5-Cl-1-oxy-2-pyridyl; R_1_ = Cl; R_2_ = (6-NH_2_-2-Me-3-pyridyl)methyl	3.05	2.89
Compounds **151**–**163**			
compound	R	Exp. Log(10^3^/Ki)	Calc. Log(10^3^/Ki)
**151** ^**b**^	R = 2.2-difluoro-2-phenylethyl	1.33	1.61
**152**	R = 2.2-difluoro-2-(pyridin-3-yl)ethyl	1.47	1.33
**153**	R = 2.2-difluoro-2-(quinolin-8-yl)ethyl	2.57	2.48
**154**	R = 2.2-difluoro-2-(isoquinolin-5-yl)ethyl	2.33	2.88
**155** ^**b**^	R = phenylsulfonyl	-0.11	0.19
**156**	R = 2-methyl-2-(pyridin-2-yl)propyl	1.24	1.55
**157**	R = 2-methyl-2-(pyridin-3-yl)propyl	0.89	0.42
**158** ^**b**^	R = 2.2-difluoro-2-(1-fluoronaphthalen-4-yl)ethyl	2.51	2.87
**159**	R = 2.2-difluoro-2-(pyridin-2-yl)ethyl	2.07	2.07
**160**	R = 2.2-difluoro-2-(quinolin-3-yl)ethyl	0.77	1.02
**161**	R = 2-(5-chloropyridin-2-yl)-2.2-difluoroethyl	2.75	2.55
**162** ^**b**^ ^**,**^ ^**c**^	R = (1-(pyridin-2-yl)cyclopropyl)methyl	1.24	0.95
**163** ^**b**^	R = (1-(pyridin-3-yl)cyclopropyl)methyl	0.85	1.53
Compounds **164**–**177**			
compound	R. R_1_. R_2_	Exp. Log(10^3^/Ki)	Calc. Log(10^3^/Ki)
**164**	R = 2.2-difluoro-2-phenylethyl; R_1_ = CN; R_2_ = H	2.64	2.16
**165**	R = 2.2-difluoro-2-phenylethyl; R_1_ = Me; R_2_ = H	2.89	2.31
**166**	R = 2-(4-fluorophenyl)ethyl; R_1_ = Me_;_ R_2_ = H	1.89	1.84
**167**	R = 2-(3.4-difluorophenyl)ethyl; R_1_ = Me_;_ R_2_ = H	1.33	1.55
**168**	R = 2-(2.4-difluorophenyl)ethyl; R_1_ = Me; R_2_ = H	1.82	1.88
**169**	R = 2-(4-trifluoromethylphenyl)ethyl; R_1_ = Me; R_2_ = H	1.33	1.82
**170** ^**b**^	R = 2-(4-methoxyphenyl)ethyl; R_1_ = Me_;_ R_2_ = H	1.96	1.63
**171**	R = 2-(3.4-dimethoxyphenyl)ethyl; R_1_ = Me_;_ R_2_ = H	0.92	1.56
**172** ^**b**^	R = 2-(4-ethylphenyl)ethyl; R_1_ = Me_;_ R_2_ = H	1.18	1.23
**173**	R = 2-(5-indanyl)ethyl; R_1_ = Me_;_ R_2_ = H	1.36	1.64
**174**	R = 2-(1-naphthyl)ethyl; R_1_ = Me_;_ R_2_ = H	1.92	1.76
**175** ^**b**^	R = 2.2-diphenylethyl; R_1_ = Me_;_ R_2_ = H	2.17	1.67
**176**	R = 2.2-difluoro-2-phenylethyl; R_1_ = Cl_;_ R_2_ = H	2.77	2.25
**177** ^**c**^	R = 2-(4-methylphenyl)ethyl; R_1_ = Me_;_ R_2_ = Me	1.43	1.53

^a^ QSAR outliers.

^b^ test set compounds.

^c^ compounds selected for MM-GBSA calculations

### Docking

The use of docking has extended in the last decades because successful applications to research in medicinal chemistry. Typically, docking programs are able to closely reproduce the binding orientations of the inhibitors in complexes for which crystallographic data are available [25–27]. Docking was performed using Glide [28], which is part of the Maestro 9.0 software suite [24]. Protein coordinates were extracted from the crystal structure of the thrombin–inhibitor complex with the code 1T4U in Protein Data Bank [6]; the protein structure was refined and completed using the Protein Wizard Preparation module also available in Maestro software. A grid box of 30Å × 30Å × 30Å was centered on the center of mass of the inhibitor in this crystal structure. The module LigPrep 2.4 [29] was used to assign ionization states, stereochemistries, and ring conformations of the ligands. Docking parameters were used as in previous works [30], Glide standard (SP) and extra-precision (XP) modes were used. The better docking poses for each ligand were examined according to their relative total energy scores. Among docking poses, the more energetically favorable conformation was selected by considering the total energy value.

### CoMSIA calculations

Compound structures and inhibitory activities as log(10^3^/Ki) values were considered for CoMSIA calculations. The relative alignment of the compounds to set field calculations was inside the binding site. For this, the 3D conformations previously obtained by docking simulations were used. QSAR modeling was performed using the Sybyl 7.3 software of Tripos [31]. The data set was divided into two sub data sets (143 and 34 compounds were in training and test sets respectively) for external validation process. The molecules contained in the training set were placed in a rectangular grid extended beyond 4 Å in each direction from the more external coordinates of each molecule. The interaction energies between a hypothetical atom (a sp^3^ hybridized carbon atom with +1 charge) and all compounds were computed at the defined points, using a volume-dependent lattice with 2.0 Å grid spacing. Then, partial least squares (PLS) method was applied using standard Sybyl parameters, and considering an optimal number of components determined by optimization of leave-one-out (LOO) cross-validation Q^2^ value (using SAMPLS [32] sampling method). Similarity was expressed in terms of steric occupancy, electrostatic interactions, local hydrophobicity, hydrogen bond (HB) donor, and HB acceptor properties, using a 0.3 attenuation factor in all CoMSIA applications.

### Molecular dynamics and MM-GBSA calculations

Complexes of the compounds **26**, **133**, **147**, **149**, **162**, and **177** inside thrombin active site were studied by MD simulations. All simulations were performed using the NAMD software package [33]. The initial coordinates for the MD calculations were taken from the docking experiments. To mimic the aqueous environment, an equilibrated water box with sides of 100 Å, centered on the mass center of each inhibitor, was used to solvate the thrombin-ligand system. Protein and inhibitors were described using the optimized potential for liquid simulations CHARMM force field [34] and the CGenFF force field [35], respectively. In turn, the water molecules belonging to the solvent box were described using the flexible TIP3P potential [36,37].

Energy minimization was performed on the models using conjugate gradient method (20.000 steps) to reduce any close contacts. Afterwards, the system was further equilibrated during 2.0 ns. The production run consisted of an MD simulation of 5.0 ns. In all cases, we applied a constraint to the backbone amino acids, we used the NPT ensemble at 300 K, we set a time step of 1 fs to solve the equations of motion, and we set a switched cutoff distance of 9.0 Å.

The free energy calculations were accomplished using the MM-GBSA method [38]. This method combines molecular mechanics energy and implicit solvation models at a reasonable computational cost, leading to outstanding results in several biological systems in recent years [39–41]. We applied this method to 500 snapshots extracted from the 5.0 ns production MD trajectories (explicit TIP3P water molecules and ions were removed for this).

Protein–ligand binding free energy using MM-GBSA was calculated as the difference between the energy of the bound complex and the energy of the unbound protein and inhibitor compound. The method allows for free energy decomposition into contributions originating from different types of physico-chemical interactions. Specifically, the energy is calculated for the protein–ligand complex, the ligand, and the protein, and their energies were computed using the CHARMM force field with the generalized Born implicit solvent model, in order to calculate the averaged binding free energy (Δ*G*) according to the following equation:

$$\Delta G_{binding}=\Delta E_{MM}+\Delta G_{solv}-T\Delta S$$ (1)

where Δ*E*_*MM*_ includes Δ*E*_*internal*_ (bond, angle, and dihedral energies), Δ*E*_*elect*_ (electrostatic), and Δ*E*_*vdw*_ (van der Waals) energies; Δ*G*_*solv*_ is the electrostatic solvation energy (polar and non-polar contributions). The polar contribution is calculated using the Generalized Born (GB) model, while the non-polar energy is estimated using the solvent accessible surface area (SASA). The conformational entropy change *‒T*Δ*S* can be computed by normal-mode analysis on a set of conformational snapshots taken from MD simulations, but many authors have been reported that the lack of the evaluation of the entropy is not critical for calculating the MM-GBSA (or MM-PBSA) free energies for similar systems [38,42,43]. In this work MM-GBSA calculations were also achieved in NAMD software [33]; the entropy term *‒T*Δ*S* was not calculated to reduce computational time.

## Results and Discussion

### Docking results

**Fig 2:**
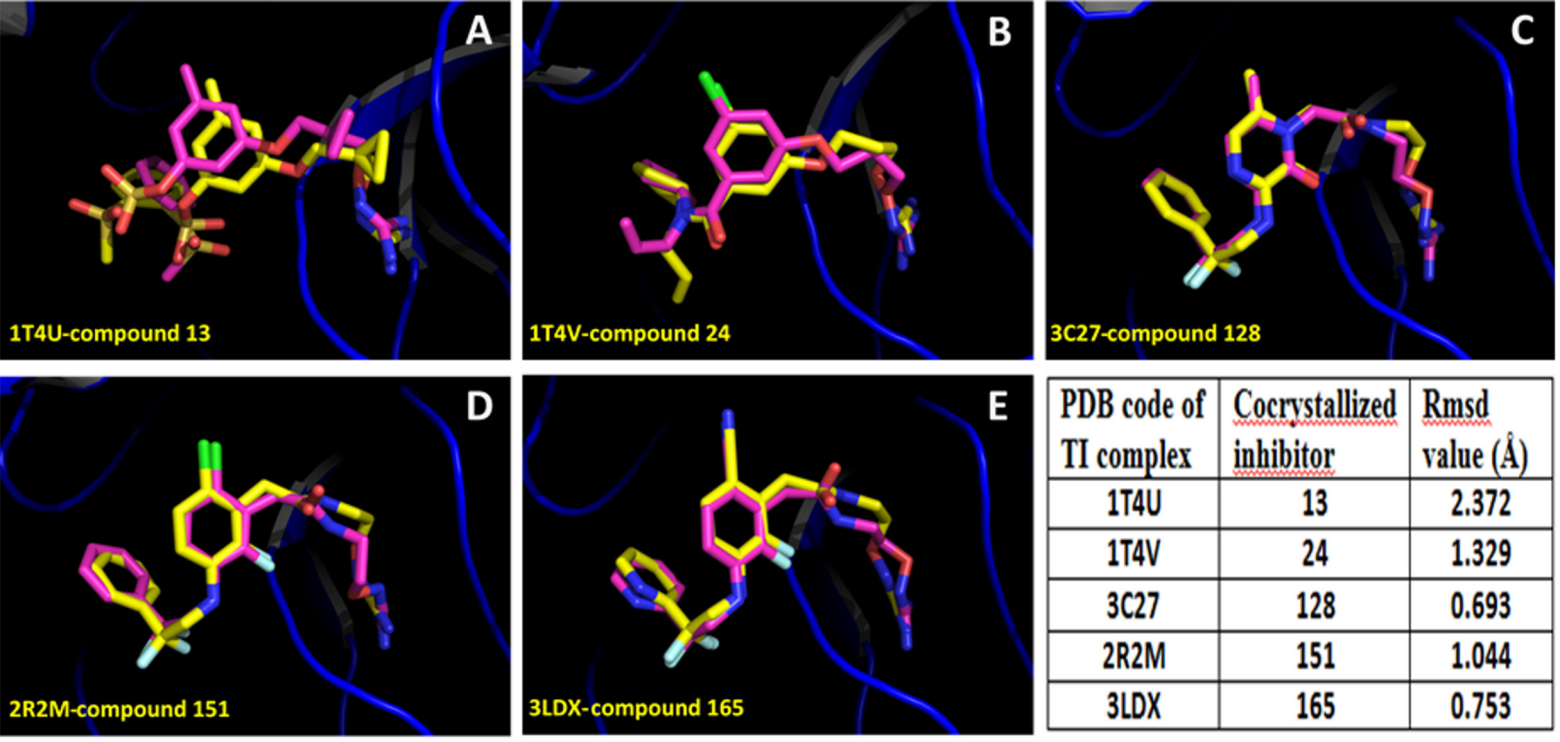
Alignment of inhibitor docked structures on inhibitor X-ray reference structures, for the TI complexes. **(A)** Compound **13** (PDB: 1T4U); **(B)** compound **24** (PDB: 1T4V); **(C)** compound **128** (PDB: 3C27), **(D)** compound **151** (PDB: 2R2M); **(E)** compound **165** (PDB: 3LDX). Crystal structures are represented in yellow, and docking results are represented in purple. Docking accuracy is reported by means of RMSD values.

First, we applied docking methodology in order to reproduce the crystal structures of thrombin-ligand complexes for compounds **13**, **24**, **128**, **151**, and **165** (accession codes in PDB: 1T4U, 1T4V, 3C27, 2R2M, and 3LDX respectively). This initial test was used to assess the quality of the docking method to reproduce known structures. In Fig 2 is shown that the docked structures fitted in an acceptable way with available inhibitor X-ray crystal structures; all the inhibitors were adequately oriented. The values of the root mean square deviation (RMSD) for the docked structures with respect to the co-crystal inhibitor structures considering all heavy atoms were < 2.0 Å in almost all the cases analyzed. Considering that 2.0 Å is the threshold value that differentiates between correct and incorrect docking solutions [44], we can state that Glide found the correct binding mode of the ligands in four of the five cases analyzed. Despite compound **13** had the RMSD value above 2.0 Å, it had the expected orientation in the thrombin binding site. The high RMSD value for compound **13** was due to a small displacement of P_2_ group, and a different orientation of the P_3_ group of the docked conformation with respect to the conformation in the crystallographic structure.

**Fig 3:**
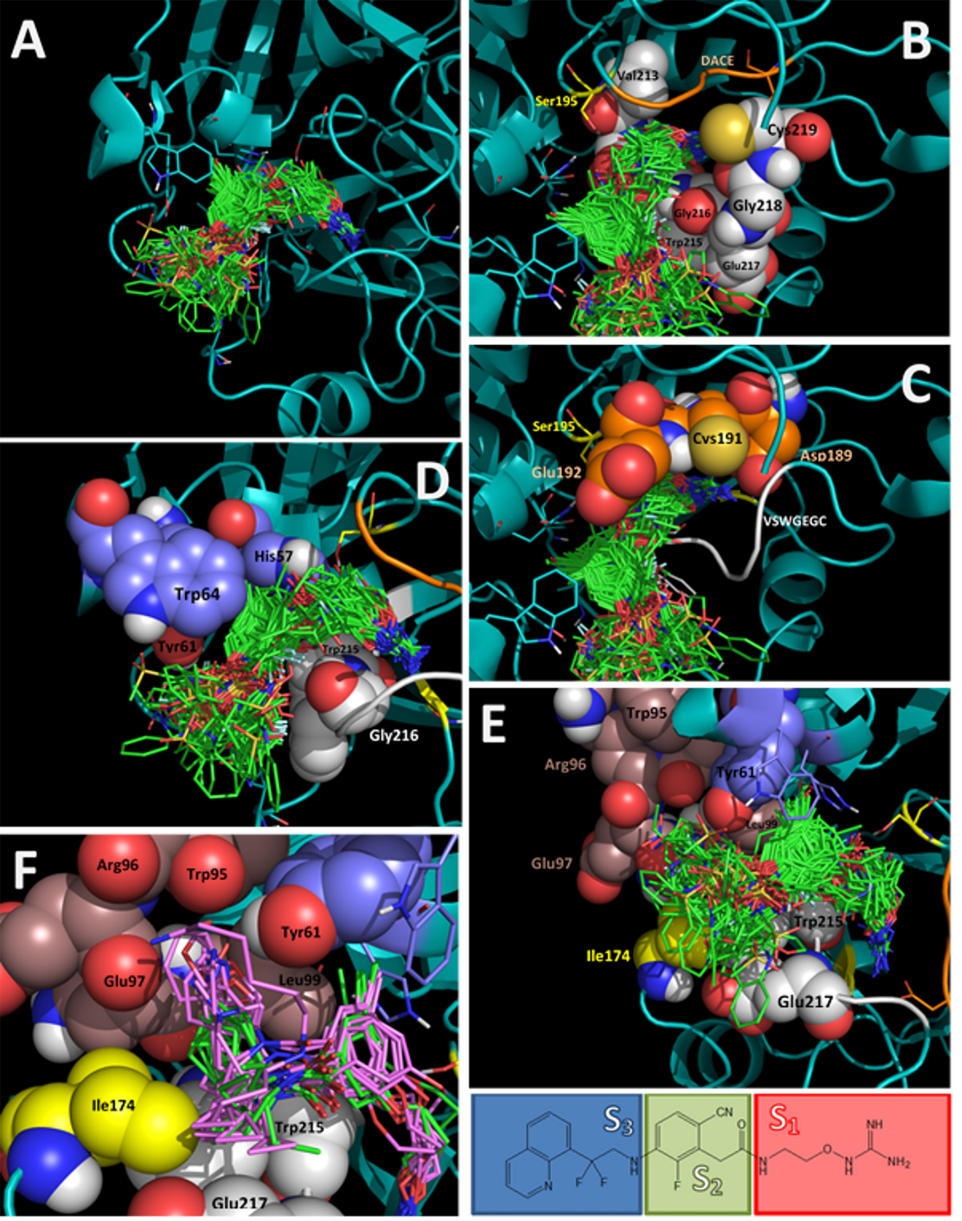
Alignment of all docked structures within the thrombin binding site (docked ligands are in green stick representation in A-E). **(A)** Full view of the binding site. **(B)** Subsite S_1_, residues of the VSWGEGC motif are represented with white spheres, DACE motif is represented as an orange loop, Ser195 is represented with yellow sticks. **(C)** Subsite S_1_, residues of the DACE motif are represented with orange spheres, VSWGEGC motif is represented as a white loop, Ser195 is represented with yellow sticks. **(D)** Subsite S_2_, residues His57, Tyr61, and Trp64 are represented with violet spheres, and residues Trp215 and Gly216 from the VSWGEGC motif are represented with white spheres. **(E**, **F)** Subsite S_3_, residues of the WRENL motif are represented with brown spheres, residues Trp215 and Glu217 from the VSWGEGC motif are represented with white spheres, and Ile174 is represented with yellow spheres. In f several compounds that have a P_1_ moiety with two branches are represented: compounds with two hydrophobic branches are represented with green sticks, and compounds with one of the branches containing a polar group are represented with pink sticks.

The remaining compounds were docked following the same docking protocol. The analysis of the docking poses obtained for all 177 ligands shows that all compounds adopt the same binding mode (Fig 3; mol2 files are in S1 File). This similar binding mode was expected since all compounds contain groups with suitable features to occupy S_1_, S_2_, and S_3_ subsites of the thrombin binding site.

The S_1_ subsite is a large cavity bounded in one part by the residues contained in the sequence VSWGEGC (Val213-Ser214-Trp215-Gly216-Glu217-Gly218-Cys219). The backbone atoms of these residues and the side chain atoms of Val213 form a semicircle controlling the shape of the S_1_ subsite (Fig 3B). The other part of the S_1_ subsite is bounded by the residues contained in the sequence DACE (Asp189-Ala190-Cys191-Glu192), where Asp189 contributes with a negative charge, Cys191 forms a disulfide bond with Cys219 from the VSWGEGC motif, and the residues Ala190, Cys191, and Glu192 contribute with their backbone atoms (Fig 3C). The hydroxyl of the residue Ser195 and the CH_2_ of Gly226 also face the subsite S_1_. In general, all compounds kept the electrostatic interactions between a HB donor group in P_1_ and the side chain carboxylate of Asp189. The backbone carbonyle groups of Gly218 and Ala190 are close to the site where interactions with Asp189 are formed; therefore they contribute with negative density to attract the P_1_ group (according to crystal structures previously reported, Gly218 can form HB with the P_1_ group). The S_1_ subsite can bound linear or cyclic groups that can adopt several conformations. The residue Ser195 (serine from the catalytic triad) has the side chain hydroxyl facing the entrance of S_1_; polar groups in this zone can interact with this residue (for instance, NH group in compounds **128**, **151**, and **165**).

The S_2_ subsite is delimited on one side by the residues Tyr61 and Trp64 from the YPPW insertion loop, and the His57 from the catalytic triad. On the other side S_2_ is delimited by the residues Trp215 and Gly216 from the motif VSWGEGC (Fig 3D). The S_2_ subsite was occupied by central aromatic group in all the docked compounds. The residues His57, Tyr61, and Trp64 form a small hole that is occupied by short substituents such as CH_3_, CN, or halogens. When bigger substituents (for instance OCH_3_ in the low active compound **91**) are present, the ligand central aromatic ring changes its orientation to place the bulky substituent out of the hole. The residue Trp215 exposes Cα and Cβ (CH and CH_2_ groups) to the S_2_ subsite; therefore it contributes to establish hydrophobic interactions in the portal between S_2_ and S_1_. Finally, the residue Gly216 exposes backbone CO and NH groups, and several TIs form HBs with them (for instance, the highly active compounds **164**‒**177** that contain the 3-aminopyrazin-2(1*H*)-one scaffold form HB with both Gly258 backbone groups).

The S_3_ subsite is an ample pocket exposed to the solvent bounded in one part by the residues contained in the sequence WRENL (Trp95-Arg96-Glu97-Asn98-Leu99). The backbone atoms of these residues and the side chain atoms of Leu99 form a wall where three CO groups (from Trp95, Arg96, and Glu97) are facing the S_3_ subsite (Fig 3E and 3F). Very close to this wall, the side chain hydroxyl group of Tyr61 faces to the S_3_ subsite. The other part of the S_3_ subsite is delimited by the hydrophobic chemical groups of the side chains of the residues Ile174, Trp215, and Glu217. Interestingly, S_3_ subsite has a hydrophilic wall (backbone CO groups of Trp95, Arg96, and Glu97, and side chain hydroxyl group of Tyr61) and a hydrophobic wall (side chains of the residues Leu99, Ile174, Trp215, and Glu217). Docking results show that the S_3_ subsite tolerates the presence of different aromatic groups. Very hydrophobic P_3_ groups were located close to the hydrophobic wall and groups with some polarity were located close to the hydrophilic wall. Very large P_3_ groups were also oriented towards the hydrophobic wall because a bigger available space in this area. For compounds that have a P_1_ moiety with two branches (compounds **24**‒**77**) we found that branches are distributed according to polarity. If the two branches are hydrophobic, both groups are located close to the hydrophobic face (compounds in green in Fig 3E), but if one of the branches contains a polar group, it is located close to the hydrophilic face and the other branch is located close to the hydrophobic face (compounds in pink colour in Fig 3E).

### CoMSIA results

CoMSIA models were carried out to provide information about the structural features affecting the thrombin inhibitory activity of the compounds under study. The use of conformations aligned using the binding site (obtained by docking) allows identifying the relevant pharmacophoric features required to best match in the binding site [45]. In this sense, the best model reported below accounts for the desired features that characterize the most potent TIs.

Firstly, the CoMSIA models were developed by including one field, and then, these fields were combined and the statistical quality of hybrid models was analyzed by considering Q^2^ values [46]. According to this analysis, we detected that compounds **5**, **18**, **33**, **90**, and **140** presented large residuals in all models; in this sense, they were excluded as outliers. Outliers are those compounds that have unexpected biological activities and are unable to fit in a QSAR model [47].

**Fig 4:**
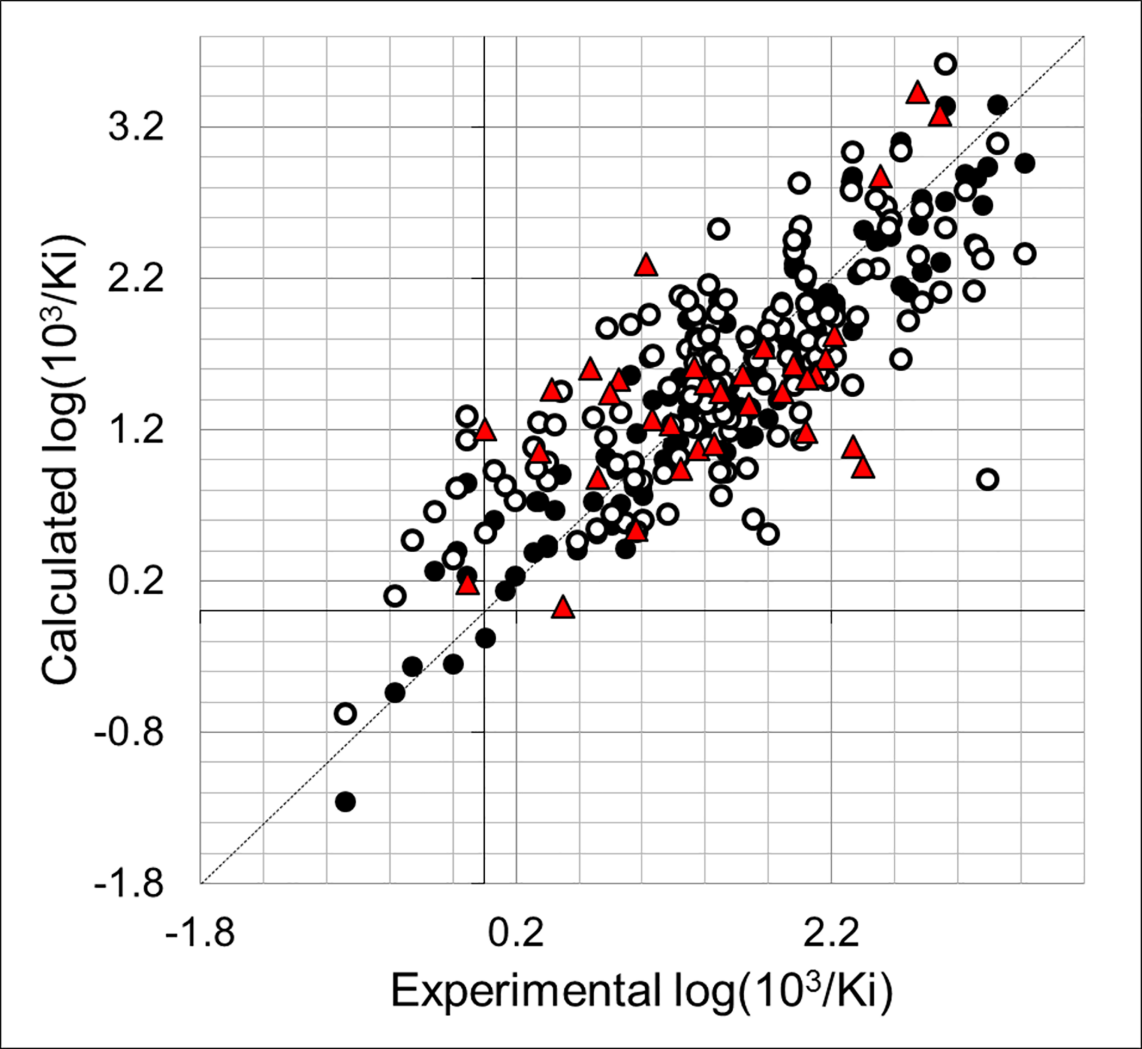
Scatter plot of the experimental activities versus predicted activities for model CoMFA-SDA: (●) training set predictions (○) LOO cross-validated predictions (red ▲) test set predictions.

The list of CoMSIA models using different fields, without considering outlier compounds, is presented in Table 2. All the combinations were tested but only the more relevant ones are reported in Table 2 (models including one field, models including two fields with Q^2^ > 0.2, models including three or more fields with Q^2^ > 0.3. The internal predictability of the models was the criterion that was used to select the best QSAR model. We observed that CoMSIA models using one field were statistically unacceptable (Q^2^ < 0.5). The analysis of the hybrid models yielded the model CoMSIA-SDA with the best Q^2^ value of 0.543. This model combines steric, HB donor, and HB acceptor fields. We included other fields and we observed that it does not produce an improvement in the internal validation of the model CoMSIA-SDA, since models including more fields had lower Q^2^ values (Table 2). The model CoMSIA-SDA was derived by using eight components and showed contributions of the steric field of 18.6%, HB donor field of 35.0%, and the HB acceptor field of 46.4%. In addition, it explains 87.1% of the variance, has a low standard deviation (s = 0.322), and a high Fischer ratio (F = 109.20). The predictions of log(10^3^/Ki) values for the 138 TIs from the training set using model CoMSIA-SDA are shown in Table 1. The correlation between the calculated and experimental values of log(10^3^/Ki) (from training and LOO cross-validation) is shown in Fig 4. Plots of the LOO cross-validation predictions reveal that the proposed model is able to discriminate between the most active and the less active compounds. Considering that the CoMSIA approach was applied to a big dataset, we consider that the value of Q^2^ = 0.543 reflects that there is no redundancy in the training set since each member of the training set is important for the model [48].

**Table 2:** Stepwise development of CoMSIA models by using SAMPLS and different field combinations.

							Fraction of fields included in the model				
	NC	R^2^	s	F	Q^2^	s_CV_	Steric	Electrostatic	Hydrophobic	HB donor	HB acceptor
CoMSIA-S	4	0.577	0.576	45.30	0.182	0.800	1				
CoMSIA-E	4	0.582	0.572	46.37	0.219	0.782		1			
CoMSIA-H	3	0.598	0.559	66.38	0.277	0.749			1		
CoMSIA-D	7	0.499	0.634	18.50	0.261	0.769				1	
CoMSIA-A	7	0.695	0.494	42.32	0.304	0.747					1
CoMSIA-SE	5	0.716	0.473	66.63	0.259	0.764	0.252	0.748			
CoMSIA-SH	3	0.635	0.533	77.63	0.295	0.740	0.250		0.750		
CoMSIA-SD	8	0.743	0.455	46.73	0.291	0.756	0.356			0.644	
CoMSIA-SA	7	0.814	0.386	81.33	0.508	0.628	0.288				0.712
CoMSIA-EH	2	0.486	0.634	62.20	0.319	0.725		0.568	0.432		
CoMSIA-ED	5	0.658	0.520	50.72	0.276	0.756		0.600		0.400	
CoMSIA-EA	7	0.825	0.375	87.33	0.410	0.687		0.519			0.481
CoMSIA-HD	3	0.600	0.558	66.99	0.322	0.726			0.543	0.457	
CoMSIA-HA	6	0.806	0.393	90.43	0.328	0.731			0.447		0.553
CoMSIA-SHD	5	0.741	0.452	75.63	0.345	0.719	0.168		0.448	0.384	
CoMSIA-SHA	6	0.825	0.373	102.82	0.395	0.693	0.155		0.355		0.490
**CoMSIA-SDA**	**8**	**0.871**	**0.322**	**109.20**	**0.543**	**0.610**	**0.186**			**0.350**	**0.464**
CoMSIA-SHDA	7	0.865	0.329	118.66	0.424	0.679	0.117		0.270	0.273	0.340
CoMSIA-ALL	7	0.878	0.312	133.87	0.439	0.670	0.085	0.262	0.198	0.201	0.254

NC is the number of components from PLS analysis; R^2^ is the square of the correlation coefficient; S is the standard deviation of the regression; F is the Fischer ratio; Q^2^ and S_cv_ are the correlation coefficient and standard deviation of the leave-one-out (LOO) cross-validation, respectively. The best model is indicated in boldface.

We also used model CoMSIA-SDA to predict the TI inhibitory activities of the test set compounds. The values are given in Table 1 and correlation between the calculated and experimental values are also represented in Fig 4. This analysis reveals that the proposed model also predicted adequately all the compounds in the test set.

**Fig 5:**
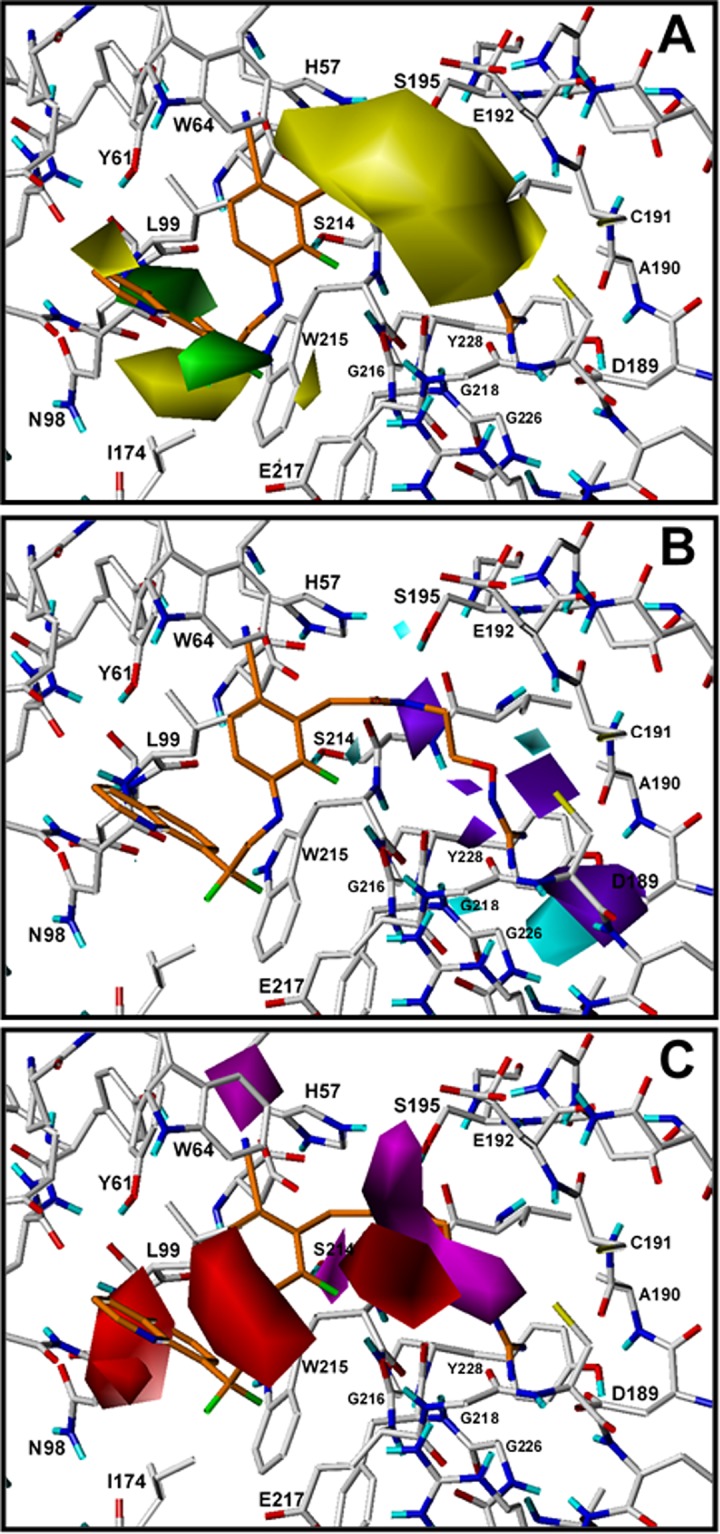
CoMSIA contour maps for TIs deriving from model CoMSIA-SDA. The amino acid residues located close to the binding pocket of thrombin are represented for comparing their position with the position of isopleths derived from the model. Compound **134** is shown inside the field. **(A)** Steric field: green isopleths indicate regions where bulky groups enhance the activity and yellow isopleths indicate regions where bulky groups disfavor the activity. **(B)**. HB donor field: cyan isopleths indicate regions where HB donors favor the activity, and purple isopleths indicate regions where HB donors disfavor the activity. **(C)** HB acceptor fields: magenta isopleths indicate regions where HB acceptors enhance the activity, and red isopleths indicate regions where HB acceptors decrease the activity.

The contour plots of the CoMSIA steric, HB donor, and HB acceptor fields are presented in Fig 5 for the best model CoMSIA-SDA. To aid in visualization, compound **134** is displayed in the maps with a superposition of the contour plots on active-site residues. The representation of contour plots and the thrombin active site in the same 3D space helps in delineating the relationship between the relevant chemical properties of the more active compounds and the chemical groups of the residues in the active site.

The colored isopleths in the map represent the 3D space where the structural properties changes are related to the changes in thrombin inhibitory potency. Green and yellow isopleths in Fig 5A indicate regions where bulky groups increase and decrease the inhibitory activity, respectively. A large region of yellow contour at S_1_ subsite suggests that bulky groups are not desired in this zone. This feature indicates that linear groups should be prioritized rather than branched groups in S_1_. Other yellow isopleths at S_3_ subsite near the residues Ile174 and Tyr61 and green isopleths near the residues Glu217 and Leu99 indicate that bulky groups extended to the more hydrophilic part of S_3_ decrease the inhibitory activity, but groups extended to the hydrophobic part of S_3_ increase the inhibitory activity. The predictions of chemical features of substituents in S_3_ according to the analysis of steric fields included in CoMSIA-SDA model are difficult since it is difficult to foresee their occupation and interactions in this wide cavity.

Cyan and purple isopleths in Fig 5B are in regions where HB donor groups favor and disfavor the activity, respectively. On the other hand, in Fig 5C magenta isopleths indicate regions where HB acceptors enhance the activity, and red isopleths indicate regions where HB acceptors decrease the activity. Interestingly, all the cyan and purple isopleths are located at S_1_ subsite. The cyan and purple isopleths near Asp189, Gly218, Gly226, and Tyr228 represent 3D spaces that the model identified that are affected by the presence of HB donor groups in the deeper region of S_1_. The purple isopleth near Ser214, the red isopleth near Gly216, and the magenta isopleth near Cys191 and Glu192 indicate that the entrance to S_1_ is affected by the presence of HB donor and acceptor groups. Two red isopleths in S_3_ indicate that HB acceptor groups are not necessary in this zone. The first one is located near Asn98 and the other is exposed to solvent between Glu217 and Trp64. The magenta isopleth in S_2_ near His57 indicates that HB acceptor groups such as CN are desired in this zone.

### Free energy calculations results

**Fig 6:**
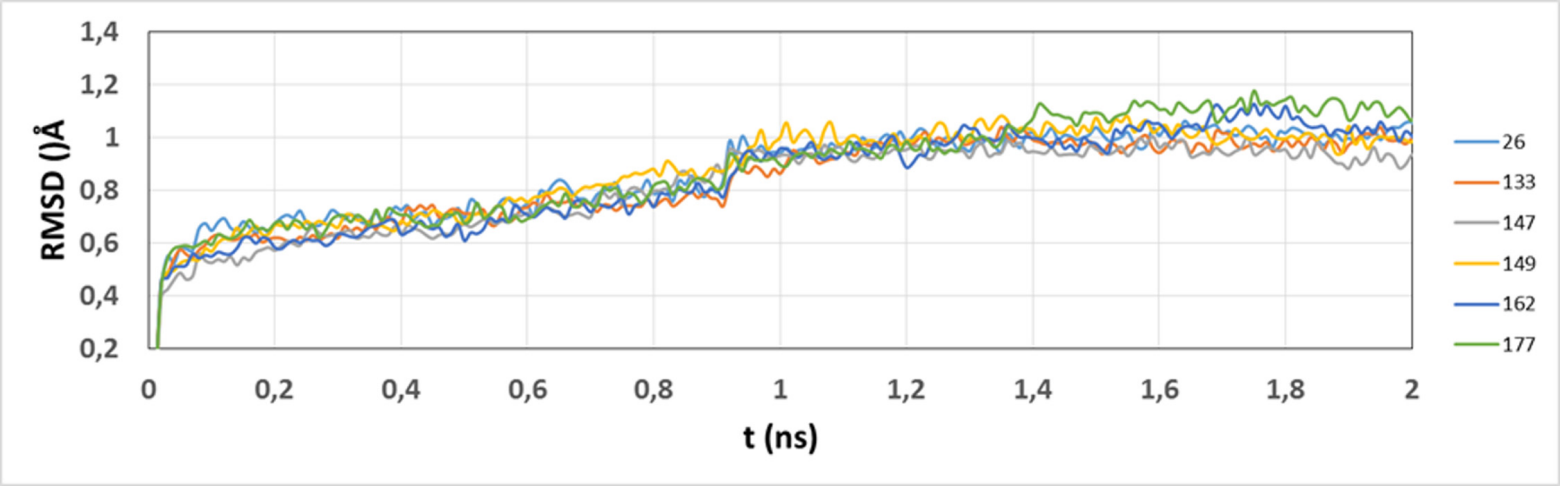
Time dependence of the RMSD for protein atoms from starting structures during equilibration process. RMSD for the studied systems are represented in colors indicated at the right.

Compounds **26**, **133**, **147**, **149**, **162**, and **177** were selected as a sample for deriving free energy calculations using MM-GBSA method. The study includes a previous conformational sampling using MD simulations to consider averaged properties and to get more realistic conditions. Stability of the MD trajectories using the RMSD of the positions for all the protein atoms as a function of simulation time was evaluated; RMSD was almost constant after the equilibration process (2.0 ns) for all the systems (Fig 6).

The above mentioned compounds were selected from data set having in mind the differences in their activities against thrombin and their differences in the substitution and structural characteristics of the groups located at S_1_, S_2_ and S_3_. In this sense, MM-GBSA paradigm allowed finding the role of physico-chemical terms in the binding potency when different groups located at S_1_, S_2_, and S_3_ are changed. Guanidinooxy groups as substituent of N-ethylacetamide or n-propanol, and 2-amino-6-methylpyridin-5-yl groups were considered in S_1_. Methyl, Cl, F, CN, and oxo groups were considered in the aromatic ring in S_2_. 2,2-Difluoro-2-arylethyl, (1-arylcyclopropyl)methyl, arylethyl, cyclopentyl groups were considered as chemical features in S_3_.

MD allows analyzing the effect of the water media, the movement of the ligand inside the binding site, the movement of the residues in the binding site, and the stability of HBs. In general, from the analysis of all the simulations, it is possible to identify different water characteristics in the pockets S_1_, S_2_, and S_3_. There are many water molecules in S_3_ which is the more exposed site to the water media, most of these molecules move freely and the other ones have occasional interactions with polar groups forming HBs. On the other hand, there are no water molecules in S_2_ during MD simulations. Finally, there are some water molecules at S_1_ trapped in HB networks formed between the ligands and the enzyme; these water molecules have a very limited movement. Interestingly, it is easy to differentiate S_1_, S_2_, and S_3_ pockets according to the distinct water dynamical behavior, and this is highly related to the TI characteristics in each pocket disclosed by docking analysis. The dynamic of the studied ligands using MD simulations revealed that groups at S_1_ form stable HB interactions, groups at S_2_ are anchored in a hydrophobic cage, and groups at S_3_ have a free movement.

**Fig 7:**
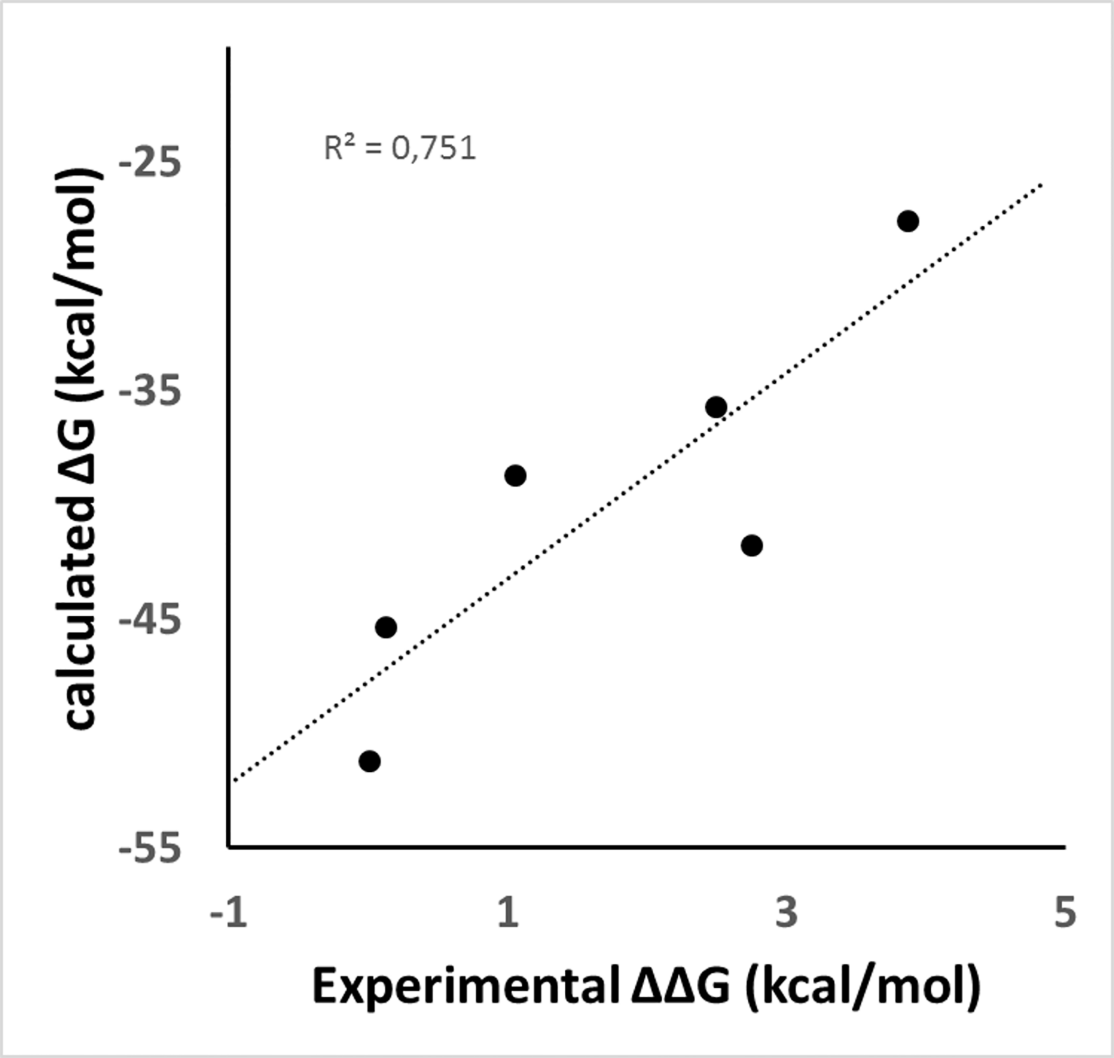
Correlation between experimental ΔΔG (calculated from Ki values of compounds) and calculated ΔG values using MM-GBSA. Experimental ΔG was calculated from Ki values of compounds, and ΔΔG was calculated using ΔG of compound **133** as reference.

The MM-GBSA free energy calculations were employed to get quantitative estimates for the binding free energies of the inhibitors inside the thrombin active site. We found a high correlation (R^2^ = 0.751, Fig 7) between calculated ΔG values and the experimental ones (the experimental values were expressed as ΔΔG values with respect to the most active selected compound **133**). However, only six compounds were considered; due to reduced data, we cannot conclude that MM-GBSA was able to explain the trend of the modeled TIs, but we can analyze the free energy components such as van der Waals (VDW), electrostatic, and solvation contributions to give detailed molecular information about the selected systems. The purpose is to evaluate the role of the physico-chemical features when different substituents are presented. The results of the predicted ΔG values and the physico-chemical components ΔE_vdw_, ΔE_elect_, and ΔG_solv_ for the complexes were summarized in Table 3. To get a better view on which energy terms have more impact on the inhibitory potency, these individual energy components were compared. From Table 3, it can be seen that ΔE_vdw_ has the major favorable contribution to the total free energy, but there is no big difference among this value for different complexes. In this sense, the VDW energy term is not primarily responsible for differentiating the binding affinity of the selected TIs. However, some of the most negative ΔE_vdw_ values were obtained for the most active compounds **133** and **149**. The solvation term also has the same effect in most of the complexes with a favorable contribution to the global free energy. Strangely, this term is positive for the most active compound **133**. On the contrary, the electrostatic term was only favorable for compound **133**, and the worst contribution of this term was for the less active compound **26**.

**Table 3:** Predicted MM-GBSA free energies (kcal/mol) and individual energy terms of the thrombin-inhibitor complexes for selected compounds.

compound	ΔG_calc_	ΔG_vdw_	ΔG_elect_	ΔG_solv_	ΔΔG_exp_ ^a^
**26**	-28.06 ± 0.10	-47.05 ± 0.10	24.65 ± 0.08	-5.65 ± 0.28	3.86
**133**	-62.74 ± 0.20	-51.38 ± 0.19	-15.33 ± 0.19	3.97 ± 0.32	0.00
**147**	-41.58 ± 0.13	-46.91 ± 0.13	10.80 ± 0.14	-5.47 ± 0.41	1.05
**149**	-45.05 ± 0.14	-59.34 ± 0.11	19.91 ± 0.14	-5.63 ± 0.39	0.12
**162**	-41.90 ± 0.22	-40.90 ± 0.23	4.58 ± 0.25	-5.58 ± 0.69	2.74
**177**	-35.74 ± 0.22	-52.54 ± 0.14	23.02 ± 0.26	-6.22 ± 0.62	2.49

a Experimental ΔΔG was calculated using ΔG of compound **133** as reference.

The analysis of the MM-GBSA terms indicates that not any physico-chemical property has a preponderant role in the potency of TIs. It is expected that this behavior could be maintained if we consider the remaining compounds considering the complexity of the thrombin binding site in S_1_, S_2_, and S_3_ pockets. We previously observed that these pockets have very different characteristics; therefore, each of them could be modulated considering different physico-chemical features. However, the binding affinity can be the result of the combination of the particular effect of these features in each pocket.

## Conclusions

In this work, we report a theoretical study on drug design area for thrombin inhibitors (TIs). We selected a data set of 177 compounds with diverse groups at motifs P_1_, P_2_, and P_3_, and explored the 3D positioning of them inside the thrombin active site pockets S_1_, S_2_, and S_3_ by docking experiments. Our approach reproduced the previously reported position of compounds **13**, **24**, **128**, **151**, and **165** inside thrombin’s active site. The remaining compounds were oriented in a similar manner. The interactions established for all compounds within thrombin binding site were carefully described.

Additionally, predictive QSAR models were built by using CoMSIA method. We found that CoMSIA-SDA including 138 compounds in the training set was the best among the explored models. This model included steric, HB donor, and HB acceptor fields. It was validated by LOO cross-validation and then it was used in the prediction of an external set which contains 34 compounds that were not included during the training process. The CoMSIA model could be used to predict novel candidates; moreover, the interpretation of the CoMSIA fields makes it possible to draw conclusions concerning the most appropriate features for novel analogues.

Finally, six compounds were selected and were subjected to MD and free energy calculations using MM-GBSA method. The dynamical behavior of the systems were revealed by MD; it was interesting to note that water molecules have completely different dynamical behavior in pockets S_1_, S_2_, and S_3_, which is related to the desired characteristics of motifs P_1_, P_2_, and P_3_ of TIs. The free energy calculations using MM-GBSA allowed studying the role of physico-chemical parameters such as van der Waals interactions, electrostatic interactions, and solvation. It was found that not any component was preponderant in the study of the differential potency of inhibitors.

## Supporting Information

S1 File Docking poses obtained for the TIs in mol2 format. (ZIP)
